# The BMP signaling pathway enhances the osteoblastic differentiation of bone marrow mesenchymal stem cells in rats with osteoporosis

**DOI:** 10.1186/s13018-019-1512-3

**Published:** 2019-12-23

**Authors:** Bin Zhao, Gengyan Xing, Aiyuan Wang

**Affiliations:** 1 Institute of Orthopaedics, Chinese PLA General Hospital, Beijing Key Lab of Regenerative Medicine in Orthopaedics, Key Lab of Musculoskeletal Trauma & War Injuries, 28 Fuxing Road, Haidian District, Beijing, 100853 People’s Republic of China; 2 Department of Orthopaedic, General Hospital of Chinese Armed Police Forces, 69 Yongding Road, Haidian District, Beijing, 100039 People’s Republic of China

**Keywords:** BMP signaling pathway, BMP-2, Smad1, Osteoporosis, Bone marrow mesenchymal stem cells, Osteoblastic differentiation, Bone mineral density, ALP

## Abstract

**Background:**

This study was conducted with the aim of exploring the effect of the BMP signaling pathway on osteoblastic differentiation in rat bone marrow mesenchymal stem cells (rBMSCs) in rats with osteoporosis (OP).

**Methods:**

The bilateral ovaries of female SD rats were resected for the establishment of a rat OP model. The osteoblastic differentiation of isolated rBMSCs was identified through osteogenic induction. Adipogenetic induction and flow cytometry (FCM) were used to detect adipogenic differentiation and the expression of rBMSC surface markers. The rBMSCs were grouped into the blank group, NC group, si-BMP2 group, and oe-BMP2 group. The expression levels of key factors and osteogenesis-related factors were determined by Western blot and quantitative real-time polymerase chain reaction (qRT-PCR). The formation of calcified nodules was observed by alizarin red staining. ALP activity was measured by alkaline phosphatase staining.

**Results:**

The rats with OP had greater weight but decreased bone mineral density (BMD) than normal rats (all *P* < 0.01). The rBMSCs from rats with OP were capable of osteoblastic differentiation and adipogenic differentiation and showed high expression of CD44 (91.3 ± 2.9%) and CD105 (94.8 ± 2.1%). Compared with the blank group, the oe-BMP2 group had elevated BMP-2 and Smad1 levels and an increase in calcified nodules and ALP-positive staining areas (all *P* < 0.05). Moreover, the expression levels of Runx2, OC, and OPN in the oe-BMP2 group were relatively higher than those in the blank group (all *P* < 0.05). The findings in the si-BMP2 group were opposite to those in the oe-BMP2 group.

**Conclusion:**

BMP signaling pathways activated by BMP-2 can promote the osteoblastic differentiation of rBMSCs from rats with OP.

## Background

Osteoporosis (OP), as a disease with escalating public health implications, is characterized by a reduction in bone mass and is the most common cause of fracture among senior populations [1, 2]. No typical symptoms of OP are usually demonstrated until a bone broken occurs; for instance, in the UK, approximately 536,000 new fragility fractures occur each year, among which there are 79,000 hip fractures, 66,000 clinically diagnosed vertebral fractures, 69,000 forearm fractures, and 322,000 other fractures [3]. Data suggest a relationship between OP and other diseases. For instance, a positive association between OP and periodontal disease [4] and a possible correlation between cardiovascular disease and OP [5] were reported. Generally, to prevent OP, bone mineral density (BMD) testing is recommended to detect bone loss and microarchitectural deterioration of bone tissue, which may be due to estrogen deficiency or vitamin D deficiency [6, 7]. In addition, to maintain the balance of bone integrity and function, the coordination of osteoblasts and osteoclasts is required, with the former being responsible for bone formation and the latter for bone resorption [8]. Therefore, the investigation of the imbalance in the activity of osteoblasts is of great value in the treatment of OP, as many bone diseases reflect imbalances in the activity of osteoblasts and osteoclasts, leading to abnormal bone mass [9].

In addition, inflammation in the bone microenvironment stimulates osteoclast differentiation [10], which may indicate the role of inflammation in OP. In addition to the effect of inflammation on OP, macrophages are recognized as being involved in bone physiology and pathology by regulating the expression of a wide range of regulatory molecules, including BMP-2 and transforming growth factor β (TGF-β) [11]. The TGF-β and BMP signaling pathways have been shown to play fundamental roles in both embryonic skeletal development and postnatal bone homeostasis [12]. Interestingly, many BMPs, including BMP-2, are believed to be potential osteogenic agents in fracture healing [13, 14]. The involvement of BMP in bone formation and osteogenesis is demonstrated by the fact that the formation of bone by osteoblast lineage cells and their principal functional activities involve complex regulation for the induction of osteogenesis, in which signals or factors including BMPs, Wnt ligands, hormones, growth factors, and cytokines are involved [15]. For instance, the activation of BMP receptors induces the differentiation of osteoblasts, resulting in the increased potential for bone formation. Nevertheless, the effect of BMP on osteoblastic differentiation in OP has been less well elucidated. In the current study, by the application of the OP rat model, we aimed to explore the effect of the BMP signaling pathway on the osteoblastic differentiation of rat bone marrow mesenchymal stem cells (rBMSCs) in rats with OP.

## Methods

### Animals for experiments

Approximately 24 Sprague-Dawley (SD) female rats without reproductive history were purchased from the Shanghai Laboratory Animal Center (SLAC) under license no. SCXK (hu) 2007-0005. The rats were aged from 4 weeks to 6 weeks and weighed 220–260 g. Before any experiments were performed, all rats were fed in the animal house and maintained at 18–28 °C for 7 days to adapt them to the environment. The humidity in the animal house was controlled at 40–70%. The air cleanliness was 100,000, and the bacterial colony population did not exceed 12.2 per container. The rats were fed in conditions in which the noise was not louder than 60 dB and the light exposure time was 12 h a day, and they were given free access to water and food; the disinfection measures and ventilation were regulated. One week later, 12 rats were randomly chosen for the establishment of the OP model, while the remaining 12 rats were grouped into the normal group.

### Establishment of the OP model

The 12 rats received intraperitoneal anesthesia with 3% pentobarbital sodium (Sigma, America). An incision was made in the abdomen to expose the abdominal cavity. Then, the lateral ovaries were resected prior to hemostasis and wound suture. Then, 80 × 10^4^ U penicillin (Shanghai Asian Pioneer, batch no. S100824) was injected into each rat twice a day for 3 days. Then, the rats were housed for 3 months with free access to food and free activities. The general information for the rats, including weight, was recorded. The bone mineral density (BMD) of the rats in both the model group and the normal group was measured using micro-CT (SCANCO) to verify the establishment of the OP model.

### Isolation and purification of rBMSCs

The OP rats were euthanized by dislocating their necks. Then, the rest of the body was soaked in 75% ethanol for 5 min, and the mandible was transferred into precooled Hank’s solution under unsterile conditions. To separate the mandible from the muscle, the muscle of the mandible was removed, after which the marrow in the mandible was collected. The cells were then gently agitated for 45 s and then filtered using a 200 mesh net. The liquid penetrating the net was then collected into a 10 mL EP tube for centrifugation at 1000 r/min for 10 min to remove the supernatant. Routine cell culture was conducted after cells were resuspended and inoculated into a culture flask at a density of 3 × 10^4^/mL. The culture medium was initially changed at an interval of 24 h and then at an interval of 3 days. Cell growth was observed under an inverted microscope. The primary rBMSCs were purified for the preparation of the cell suspension, which was then transferred into a 96-well plate. The single cells in each well were marked, and the culture medium was replaced 3 days later. After the cell density reached 50%, the cell suspension was inoculated into a six-well plate for further culture.

### Identification of rBMSCs

Pancreatic enzymes (Sigma, America) without EDTA were used to digest the third passage of rBMSCs, which was followed by centrifugation at 1000 r/min to dispose of the supernatant and three washes with PBS. PBS was applied to the resuspended cells, and the cell density was adjusted to 6000 cells/μL in the tube. Then, antibody markers for CD105, CD34, CD44, and CD45 were added into the tube prior to incubation in a dark room at ambient temperature for half an hour. The cells were fixed with methanol after PBS washing and centrifugation and then maintained overnight in a refrigerator at 4 °C without light exposure. On the next day, the expression levels of CD105, CD34, CD44, and CD45 were measured using flow cytometry (FCM, Partec, Germany).

### Identification of the osteoblastic differentiation of rBMSCs

After the cells were cultured for 24 h until they fully adhered to the tube well, the original culture medium was replaced with osteogenic induction medium (complete culture medium with 10^− 8^ mol/L dexamethasone and 10 mmol/L sodium β-glycerophosphate, both from Sigma, America). The culture medium was changed every 2–3 days. After 2–3 weeks, the slide was removed until the mineralized nodules were exposed for alizarin red staining. The slide was first washed with PBS and then fixed with 95% ethanol. Approximately 10 min later, the slide was washed twice with distilled water and soaked with 0.1% alizarin red (pH = 8.3, Sigma, America) at 37 °C for 30 min. The osteoblastic differentiation of rBMSCs was identified with a microscope after the slide was washed twice with PBS.

### Identification of the adipogenic differentiation of rBMSCs

After the cells were cultured for 24 h until they fully adhered to the tube well, the original culture medium was replaced with adipogenic induction medium (with 10^− 6^ mol/L dexamethasone, 10 mg/L insulin (Sigma, America), 0.5 mmol/L 3-isobutyl-l-methyl-xanthine (IBMX, Sigma, America), and 100 mmol/L indomethacin). The culture medium was changed every 2–3 days. After 2–3 weeks, the slide was removed after the formation of a lipid droplet for Oil Red O staining. The slide was first washed with PBS three times, and the extra water was removed; then, the slide fixed with 10% neutral ethanol for 10 min. The slide was soaked with Oil Red O (Sigma, USA) for 10 min, followed by washing with distilled water for 10 min. The adipogenic differentiation of rBMSCs was identified with a microscope.

### Cell grouping and treatment

The well-developed rBMSCs from rats with OP from the third passage were classified into the blank group, negative control (NC) group (transfected with NC BMP-2 plasma from rBMSCs of rats with OP; GenePharma, Shanghai), si-BMP2 group (plasma from rBMSCs of rats with OP with low expression of BMP-2; GenePharma, Shanghai), and oe-BMP2 group (plasma from rBMSCs of rats with OP with the overexpression of BMP-2; GenePharma, Shanghai). Cells in the above groups were cultured with osteogenic induction medium for 14 days prior to cell collection for further experiments.

### Real-time quantitative fluorescent polymerase chain reaction

Total RNA was extracted from cells in each group according to the TRIzol method (Invitrogen). The high-quality RNA was then identified by UV analysis technology and electrophoresis on a gel containing formaldehyde. RNA (l μg) was reverse-transcribed into cDNA using AMV reverse transcriptase. PCR primers were designed and synthesized by Invitrogen (Table 1). PCR conditions were as follows: predenaturation at 94 °C for 5 min, followed by 40 cycles of denaturation at 94 °C for 40 s, annealing at 60 °C for 40 s, extension at 72 °C for 1 min, and a final extension at 72 °C for 10 min. The PCR products were electrophoresed on a sepharose gel and analyzed using OpticonMonitor3 software (BioRad). The threshold value of the lowest point of the amplification curve was manually set along the rising curve. The threshold cycle (Ct) was analyzed using the 2^−ΔΔCt^ method. The 2^−ΔΔCt^ value represented the ratio of the gene expression in the experimental group and the normal group: ΔΔCt = [Ct(target gene) − Ct(control gene)]_experimental group_ − [Ct(target gene) − Ct(control gene)]_normal group._ Experiments were conducted three times to obtain the average value. GAPDH was considered as a control.

**Table 1:** Primer sequences

Gene	Sequence
BMP-2	F: 5′-AACGAGAAAAGCGTCAAGCCFF -3′
	R: 5′- CCAGTCATTCCACCCCACAFF -3′
Smad1	F: 5′- CAGCAGAGGAGATGTTCAGGCA -3′
	R: 5′- TGCACGAAGATGCTGCTGTCAC -3′
Runx2	F: 5′- TGAGCGACGTGAGCCCGGTA -3′
	R: 5′- CGTGTGGAAGACAGCGGCGT -3′
OC	F: 5′- CCTGGCAGGTGCAAAGCCCA -3′
	R: 5′- TGCGCTTGTAGGCGTCCTGG-3′
OPN	F: 5′- TGCCTGTTCGGCCTTGCCTC -3′
	R: 5′- TCCAGGCTGGCTTTGGAACTCG-3′
GAPDH	F: 5′- CAAGTTCAACGGCACAGTCA -3′
	R: 5′- CCCCATTTGATGTTAGCGGG-3′

### Western blot analysis

Proteins from cells in each group were extracted to identify the protein concentration using a BCA kit (Boster, Wuhan). Loading buffer was then added to the proteins, which were boiled at 95 °C for 10 min. Then, 30 μg of loading buffer was added to each well, and the proteins were separated by a 10% polyacrylamide gel (Boster, Wuhan). The voltage for electrophoresis was increased from 80 V to 120 V, and the proteins were transferred to a membrane at 100 mV for 45–70 min. The membrane was transferred to PVDF, followed by blocking with 5% BSA at ambient temperature for 1 h. β-actin was considered as a control. Primary antibodies for BMP-2, Smad1, p-Smad1, Runx2, osteocalcin (OC), and osteopontin (OPN) (1: 1000, Abcam, America) and primary antibody for β-actin (1:3000, Abcam, America) were added and incubated overnight at 4 °C. The membrane was washed three times with TBST for 5 min each. Incubation with secondary goat anti-rat antibodies (Jackson, America) at ambient temperature was conducted. Then, the membrane was washed three times for 5 min each. The color of the membrane was developed using chemiluminescence reagents. A Bio-Rad Gel Dol EZ imager (GEL DOC EZ IMAGER, Bio-Rad, California, USA) was used for the color observation. ImageJ software was used to analyze the grayscale value of the target band. Experiments were conducted three times to obtain the average value.

### Alkaline phosphatase (ALP) staining

Alkaline phosphatase staining was performed 7 days after osteogenic induction in accordance with the following procedure. Cells were first fixed with ethanol for 15 min, followed by PBS washing three times and the addition of ALP staining solution (Beyotime Biotechnology) prior to incubation for 30 min in the dark until a certain degree of staining was obtained. After that, the staining solution was removed, and the cells were washed with distilled water twice. After the cells were dried, ImageJ software was used for semiquantitative analysis of the ALP staining.

### Staining of mineralized nodules in cells

The culture medium was discarded after 14 days of osteogenic induction. Then, the cells were washed twice and fixed with 10% neutral buffered formalin. Thirty minutes later, the formalin was removed, and the cells were washed in PBS twice prior to staining with 1 mL of alizarin red for 3–5 min in each well. After the staining was complete, the staining solution was removed, and the cells were observed and photographed after being washed in PBS twice. The cells in each well were incubated with 1 mL of extraction buffer containing a 8:2 ratio of 10% acetic acid solution to absolute ethyl alcohol for 30 min without light exposure. A microplate reader was used to measure the absorbance at 490 nm. The cell mineralization was then semi-quantified according to the absorbance.

### Statistical analysis

All data to be analyzed were expressed as the mean ± standard deviation (SD) and analyzed using SPSS 21.0 (SPSS, Inc., Chicago, IL, USA). The measured data that comply with the normal distribution were analyzed using a *t* test. The differences between two groups were investigated using the least significant difference (LSD), and comparisons among multiple groups were analyzed using one-way ANOVA. A difference was considered statistically significant if the *P* value was less than 0.05.

## Results

### General information on rats with OP

**Fig. 1:**
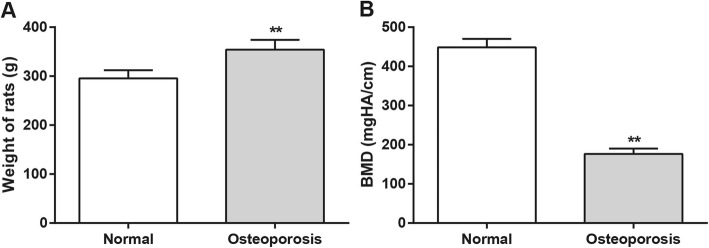
Comparison on weights and BMD of rats in OP model group and normal group. Note: **a**, weights of rats in OP model group and normal group; **b**, BMD of rats in OP model group and normal group. **Compared with rats in normal group, *P* < 0.01; OP, osteoporosis; BMD, bone mineral density; rBMSCs, rat bone marrow mesenchymal stem cells; OP, osteoporosis

The OP rat models were revived 1–2 h after model establishment and began to drink and eat 4 h later. After 5 days, the rats recovered normal activity, and their wounds recovered and showed the absence of infection and defects in the abdomen. Three weeks later, the rats with OP had increased weight and duller, grayish hair and were less active when compared with the rats in the normal group, which showed mildly increased weight, shiny hair, and normal activity. Three months later, the weights of the rats in the model group were greater than those in the normal group (*P* < 0.01) (Fig. 1a). The bone mineral density (BMD) between rats in the model group and the normal group was compared after model establishment for 3 months. The results indicated that the rats with OP had decreased BMD when compared with those in the normal group (176.4 ± 13.8 mgHA/cm VS 448.5 ± 21.6 mgHA/cm, *P* < 0.01), which indicates that the OP rat model was successfully established (Fig. 1b).

### Observation of rBMSCs from rats with OP

**Fig. 2:**
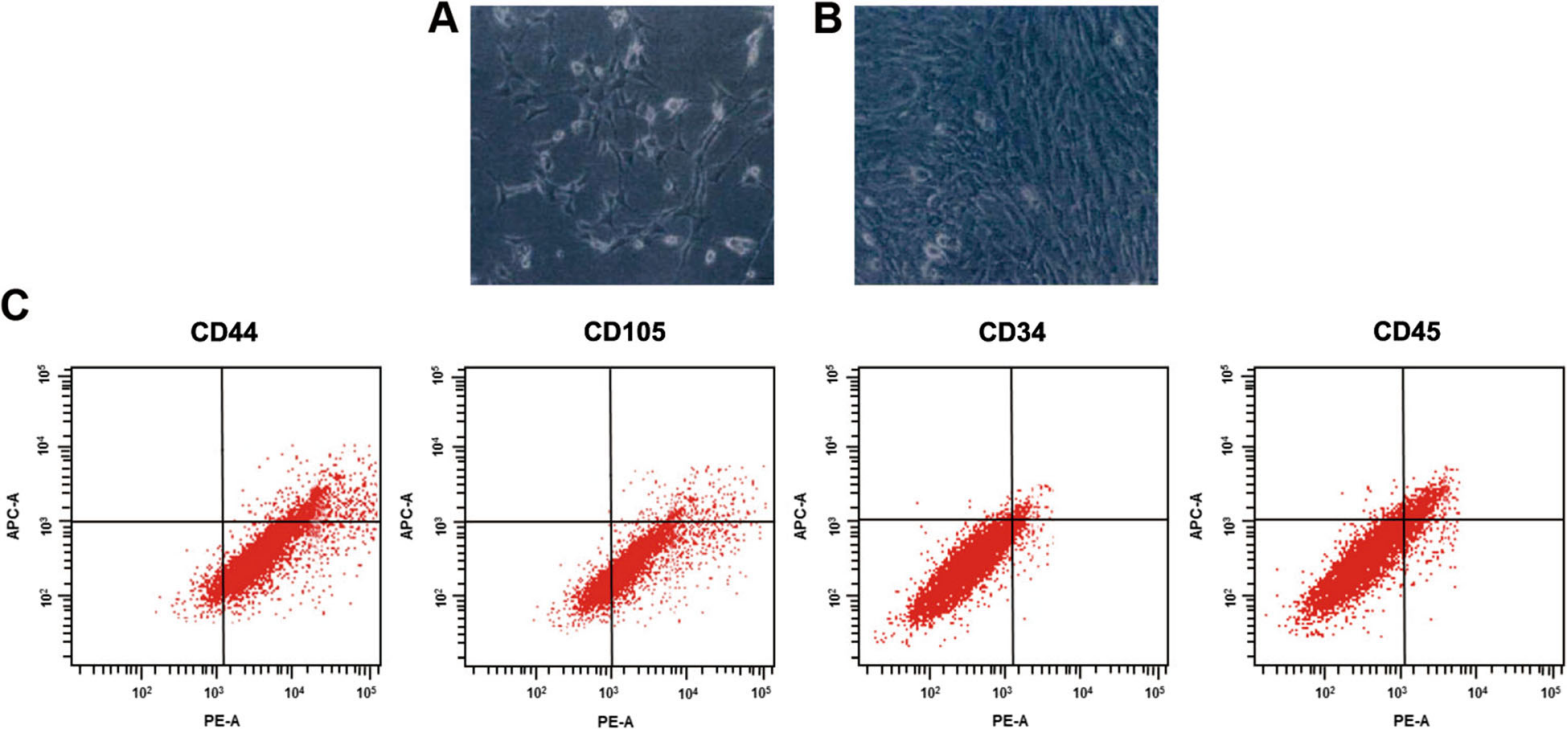
Morphological and identification of phenotypic characteristics of rBMSCs of rats with OP. Note: **a**, rBMSCs after being cultivated for 10 days; **b**, rBMSCs after being cultivated for 15 days; **c**, identification of phenotypic characteristics of rBMSCs of rats with OP; rBMSCs, rat bone marrow mesenchymal stem cells; OP, osteoporosis

The observation of the rBMSCs under an inverted microscope showed that the primary rBMSCs had grown into colonies in shuttle or triangle shapes and presented with parenchyma cells that were round-shaped and not adherent to the container wall. The cultivation of primary rBMSCs for 12–15 days was able to achieve cell fusion, and P3 cells were observed after 5–8 days (Fig. 2a, b).

### Expression of CD105 and CD44 in rBMSCs from rats with OP

FCM was used to detect the expression of CD105 and CD44 in rBMSCs from rats with OP. The P3 rBMSCs had high expression of CD44 (91.3 ± 2.9)% and CD105 (94.8 ± 2.1)%, but no expression of CD34 (3.5 ± 1.2)% and CD45 (5.2 ± 1.4)%. The results were consistent with the phenotypic characteristics of rBMSCs (Fig. 2c).

### Identification of osteogenic and adipogenic differentiation

**Fig. 3:**
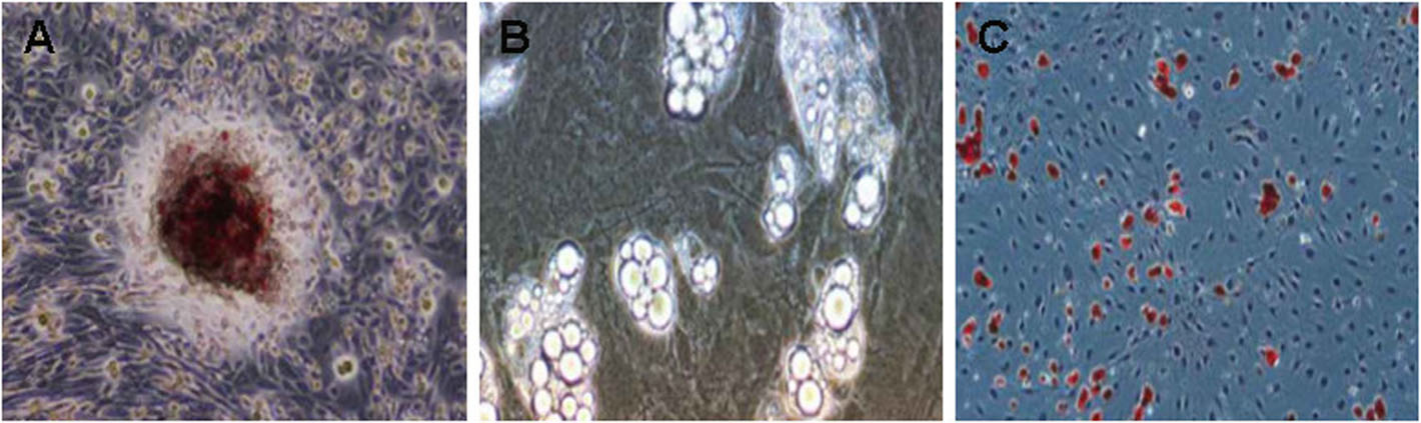
Identification of osteogenic and adipogenic differentiation in rBMSCs of rats with OP. Note: **a**, alizarin red staining after osteogenic induction for 21 days; **b**, adipogenic induction for 7 days under observation of a light microscope; **c**, oil red o staining after adipogenic induction for 14 days; OP, osteoporosis; rBMSCs, rat bone marrow mesenchymal stem cells

Alizarin red staining showed the presence of the majority of red mineralized nodules after 21 days of osteogenic induction, and the central part of each nodule was brown black, which suggests the formation of calcium nodules (Fig. 3a). Seven days after adipogenic induction, the cell synapses had shrank, and light and transparent yellow lipid droplets were found in the cytoplasm, which were first found on the cell edges (Fig. 3b). Over time, the number of lipid droplets increased, and the droplets started to merge into a large lipid droplet. Approximately 14 days later, Oil Red O staining showed red and round lipid droplets that were arranged in a moniliform in the cytoplasm (Fig. 3c).

### Expression of BMP-2 and Smad1 in rBMSCs from rats with OP

**Fig. 4:**
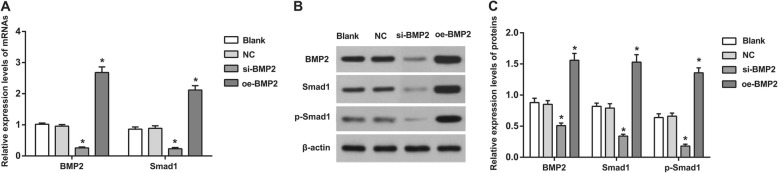
Comparisons on expressions of BMP related proteins in each group. Note: **a**, mRNA expressions of BMP related proteins; **b**, protein expression of BMP related proteins; **c**, statistical analysis of protein expression of BMP related proteins; *Compared with the blank group, *P* < 0.05

qRT-PCR and Western blotting showed that compared with the blank group, the si-BMP2 group had decreased mRNA and protein expression of BMP-2 and Smad1 as well as reduced phosphorylation of p-Smad1 (all *P* < 0.05), indicating that siRNA interference with BMP-2 can suppress the activation of the BMP signaling pathway. In contrast, the oe-BMP2 group had higher mRNA and protein expression levels of BMP-2 and Smad1 and elevated phosphorylation levels of p-Smad1 than those in the blank group (all *P* < 0.05), which implies that the overexpression of BMP-2 can activate the BMP signaling pathway. No significant difference between the NC group and the blank group was detected (all *P* > 0.05) (Fig. 4).

### Effect of BMP-2 expression on alizarin red staining

**Fig. 5:**
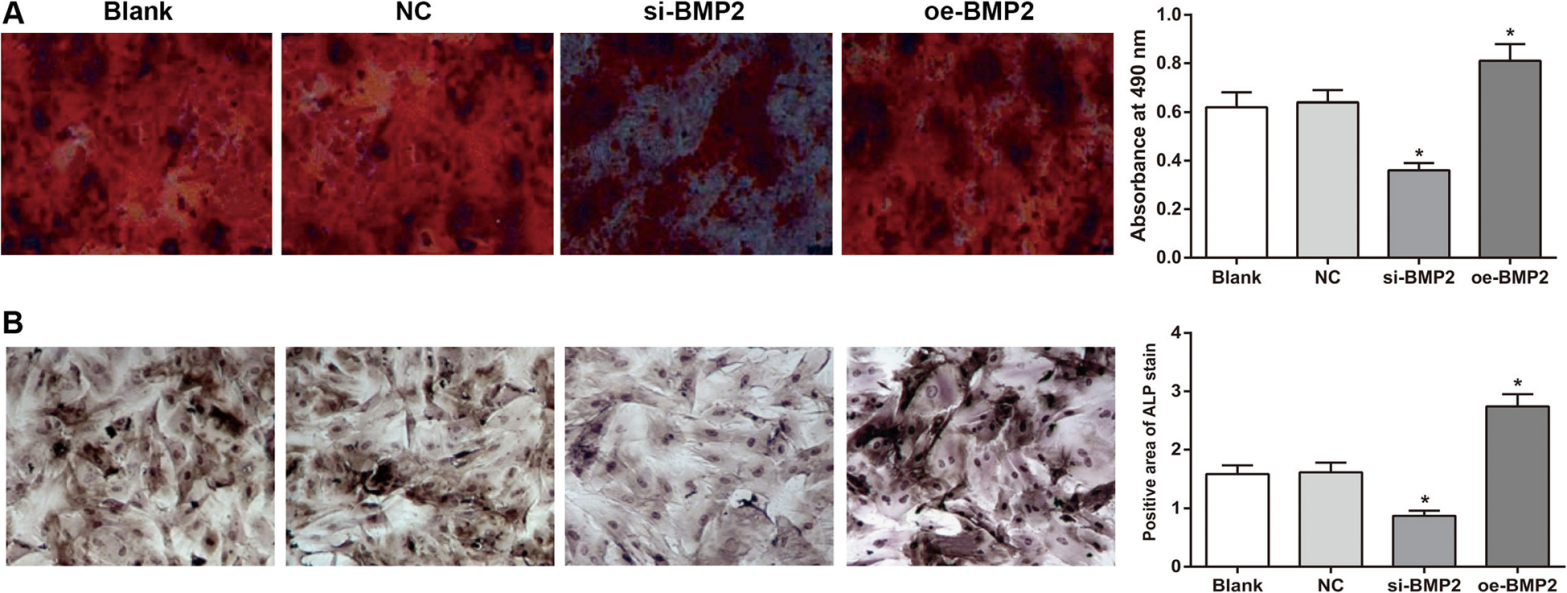
Alizarin red staining and ALP staining in each group (× 200) Note: **a**, alizarin red staining in each group; **b**, ALP staining of each group; *Compared with the blank group, *P* < 0.05

Alizarin red staining demonstrated that after 14 days of osteogenic induction, positive red nodules of various sizes were observed in each group. In comparison with the blank group, the numbers and sizes of the calcified nodules in the oe-BMP2 group were increased, while those in the si-BMP2 group were decreased (all *P* < 0.05). These results indicated that BMP-2 can promote osteoblastic differentiation of rBMSCs from rats with OP. The comparisons of the numbers and sizes of calcified nodules between the NC group and the blank group showed no significant differences (both *P* > 0.05) (Fig. 5a).

### ALP staining of each group

In contrast to the blank group, the oe-BMP2 group had a larger ALP-positive staining area in the rBMSCs (*P* < 0.05), while the si-BMP2 group had a decreased ALP-positive staining area in the rBMSCs (*P* < 0.05). No significant difference between the NC group and the blank group was detected (*P* > 0.05) (Fig. 5b).

### Expression of osteogenesis-related genes in each group

**Fig. 6:**
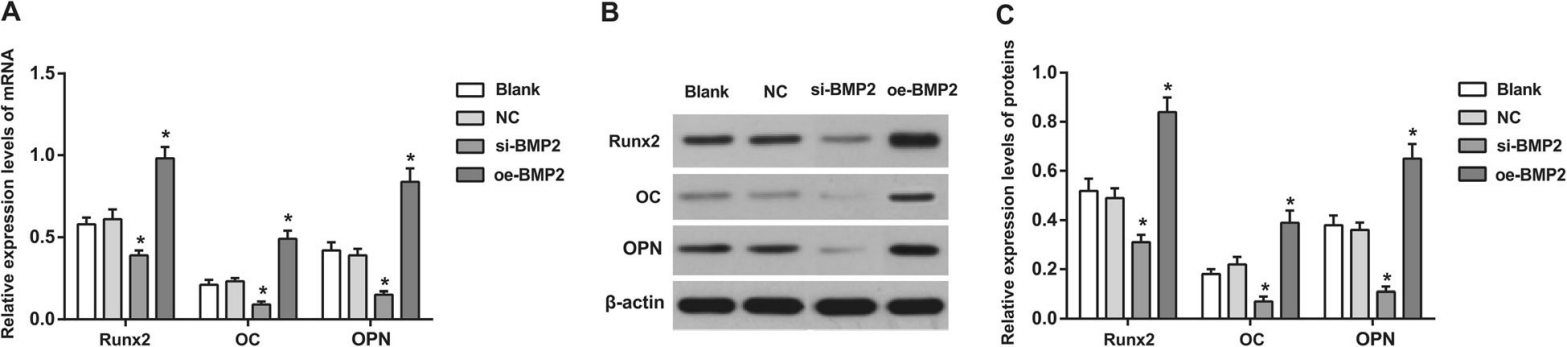
Expressions of osteogenesis related genes, Runx2, OC and OPN in each group. Note: **a**, mRNA expressions of Runx2, OC, and OPN; **b**, protein expressions of Runx2, OC, and OPN; **c**, statistical analysis of protein expressions of Runx2, OC, and OPN; *Compared with blank group, *P* < 0.05; OC, osteocalcin; OPN, osteopontin

The expression of Runx2, OC, and OPN in the blank group was not significantly different from those in the NC group (all *P >* 0.05). The si-BMP-2 group had decreased expression of Runx2, OC, and OPN, while the oe-BMP2 group had elevated expression of Runx2, OC, and OPN in comparison to the blank group (all *P <* 0.05). These results indicated that activation of the BMP signaling pathway can upregulate the expression of the osteogenesis-related genes Runx2, OC, and OPN, thus contributing to osteogenesis (Fig. 6).

## Discussion

OP, together with its main health outcome, which is fragility fractures, is a large and escalating global issue [1]. Efforts have been made to overcome this obstacle, and genetic factors are proposed to be major contributors to the pathogenesis of this disease [16]. The understanding of the genetic factors of OP is of crucial value for disease prevention [17] and for the search for novel therapies. Our results showed that OP rats had lower BMD than healthy rats, which indicated that the bone strength in OP rats was weak and partially explained the role of OP in bone fracture.

Further experiments with qRT-PCR, Western blotting, and FCM found that CD44 and CD105 were highly expressed in rBMSCs and that CD34 and CD45 were absent in rBMSCs from rats with OP. CD44 antigen is a cell-surface glycoprotein that is mainly implicated in cell-cell interactions and cell adhesion and migration in a wide variety of cell activities, including lymphocyte activation, recirculation and homing, hematopoiesis, and tumor metastasis [18]. The data indicated that high expression of CD44 plays a vital role in the initiation of malignant transformation and epithelial-mesenchymal transition (EMT)-like processes [19, 20]. CD44 also interacts with other ligands, such as osteopontin and matrix metalloproteinases (MMPs), in adult cardiac myocytes and osteoclasts [21, 22]. CD105, also referred to as ENG, END, FLJ41744, HHT1, ORW, and ORW1, predominantly participates in angiogenesis, making it an important protein for tumor growth and survival and the metastasis of cancer cells to other locations in the body [23]. The detection of CD44 and CD105 in rBMSCs implied that bone weakness and damage occurred in OP rats, which was further supported by the observation of the absence of CD34 and CD45 in rBMSCs. In a previous publication, RT-PCR of osteogenic/endothelial markers revealed the osteogenic and vasculogenic plasticity of transplanted human multipotent adipose-derived stem (hMADS) cells at the early stage of fracture healing, in which mesenchymal cell surface markers such as CD44 and CD105 were positively expressed in human multipotent adipose-derived stem cells [24]. In the same study, the proliferation and migration of hMADS cells in vitro were found to be activated by the presence of BMP-2 or vascular endothelial growth factor (VEGF).

Importantly, the investigation of the role of the BMP signaling pathway in the osteoblastic differentiation of rBMSCs suggested that the low expression of BMP-2 can suppress the activation of the BMP signaling pathway, while activation of the BMP signaling pathway can promote the osteoblastic differentiation of rBMSCs in rats with OP. Osteoporotic fractures were characterized by slow callus formation and bone healing, in which the cellular sources of bone healing are mesenchymal stem cells (MSCs) that differentiate into osteoblasts that are recruited by osteoinductive cytokines, such as BMP-2 [25, 26]. Transforming growth factor-beta (TGF-β)/bone morphogenic protein (BMP) signaling is involved in a vast majority of cellular processes [8]. Early studies suggested that BMP-2 can play a critical role in the differentiation of osteoblasts through the regulation of the Smad signaling pathway [27]. Similarly, the activation of R-Smads (Smads 1/5/8), the major transducers of BMP receptors, involves translocation in the nucleus through interactions with various transcription factors [28]. Specifically, a complex with Smad4 was generated, which was translocated into the nucleus to activate the transcription of Cbfa1/Runx2, thereby regulating bone metabolism [29]; this explained the observation of Runx-2 expression in our study. The balance between Runx2 and TGF-β/BMP-activated Smads is critical for the formation of the skeleton [8]. Since OP is a complex condition involving multiple factors, genes, and cytokines, the exact relationship between BMP and osteoblastic differentiation in rBMSCs from OP rats will require more longitudinal studies. Collectively, these results may indicate the possible mechanism involved in the effect of the BMP signaling pathway in regulating osteoblastic differentiation in OP, in which coordination with the Smad pathway is largely involved.

## Conclusion

In summary, OP is a common threat to public health and is characterized by fracture and reduced bone formation. Overexpression of BMP-2 can promote the activation of the BMP signaling pathway, which in turn may accelerate osteoblastic differentiation in rBMSCs.

## Data Availability

The datasets used and analyzed during the current study are available from the corresponding author on reasonable request.
